# A rare case of a young man with mediastinal synovial sarcoma

**DOI:** 10.1016/j.rmcr.2024.102079

**Published:** 2024-06-21

**Authors:** Intan Nurani Indrajanu, Isnin Anang Marhana, Dwi Wahyu Indrawanto

**Affiliations:** Department of Pulmonology and Respiratory Medicine, Faculty of Medicine, Universitas Airlangga – Dr. Soetomo General Academic Hospital, Surabaya, Indonesia

**Keywords:** Mediastinal synovial sarcoma, Doxorubicin ifosfamide, Pleural effusion

## Abstract

Synovial sarcoma is a rare and aggressive tumor that primarily affects soft tissues, including the mediastinum, and predominantly affects younger adults. A 23-year-old male patient with mediastinal synovial sarcoma underwent debulking surgery and received 3 cycles of doxorubicin, ifosfamide, and mesna chemotherapy. Mediastinal synovial sarcoma presents diagnostic challenges and poor prognosis. Treatment involves surgical resection, adjuvant chemotherapy, and radiotherapy. Mediastinal synovial sarcoma can be diagnosed through histopathological and immunohistochemical examination. Adjuvant chemotherapy led to a partial response, showing a decrease in tumor size and resolution of pleural effusion, demonstrating a positive interim outcome.

## 1 Introduction

Synovial sarcoma is a rare and aggressive malignant tumor that presents challenges in diagnosis and treatment. It primarily occurs in soft tissues of the extremities, often near joint capsules and tendon sheaths, but also can occur in other areas of the body, including the mediastinum [1]. Although it is known as "synovial", it does not originate from synovial tissue but rather from pluripotent mesenchymal tissue which commonly found in tendons and joint fibers [2].

Synovial sarcoma accounts for approximately 5%–10% of all soft tissue sarcomas and predominantly affects younger patients. In the thoracic region, it is most commonly found in the pleuropulmonary system, with mediastinal involvement being rare [1,3]. Studies have shown that mediastinal synovial sarcoma occurs more frequently in young adults, with a higher incidence in males [4].

Synovial sarcoma (SS) can present as one of three histological variants: monophasic, biphasic, and poorly differentiated. Immunohistochemistry is ideal for distinguishing these subtypes. SS is positive for epithelial membrane antigen (EMA), bcl-2, CD99, S-100, and vimentin and negative for myoD1 and myogenin [2]. More than 95% of SS cases have the t(X; 18)(p11; q11) translocation, resulting in SS18-SSX fusion genes [4].

Complete surgical resection is the preferred treatment for mediastinal synovial sarcoma, with adjuvant chemotherapy and radiotherapy considered for inoperable non-metastatic disease. High-dose ifosfamide with or without doxorubicin is recommended for failed cases. Adjuvant chemotherapy and radiotherapy should be considered for all patients [3,5].

The five-year survival rate for adults with synovial sarcoma has remained stable since the 1980s, with no significant advancements in treatment efficacy [1]. The prognosis for mediastinal synovial sarcoma remains poor, with an overall survival of 36 months and a five-year survival rate of 35.7%, compared to 50%–80% for primary extremity tumors. Factors contributing to this poor prognosis include advanced stage at presentation, large tumor size, difficulty in achieving complete surgical resection due to involvement of vital anatomical structures, and a high incidence of poorly differentiated subtypes. The specific gene fusion type (SYT-SSX1 vs. SYT-SSX2) does not significantly impact disease-specific survival in thoracic synovial sarcoma [3].

Systemic treatment may be necessary in some cases, but there is no consensus on the optimal systemic therapy. Surgical intervention is the primary consideration, but complete tumor removal may be challenging when vital neurovascular structures or the chest wall are involved. High-dose doxorubicin (e.g., adriamycin regimen combined with ifosfamide) is recommended for more than three cycles in high-risk patients [6]. The combination of doxorubicin-ifosfamide is also suggested as adjuvant therapy for recurrent tumors [2].

## 2 Case presentation

A 23-year-old male patient admitted to the hospital with the main complaints of shortness of breath for 2 months, worsened when lying down, cough, chest pain for 3 months, and worsening with yellowish-white sputum. The patient experienced a weight loss of approximately 5 kg in 3 months. CT scan results showed a mediastinal tumor in the right lung. Pathological examination revealed a spindle mesenchymal tumor. The patient had a history of using electronic cigarettes for about 5 years. Physical examination revealed general weakness, asymmetric chest shape, decreased tactile fremitus in the upper third of the right hemithorax, dullness on percussion in the upper third of the right hemithorax, and decreased breath sounds in the upper third of the right hemithorax.

**Fig. 1:**
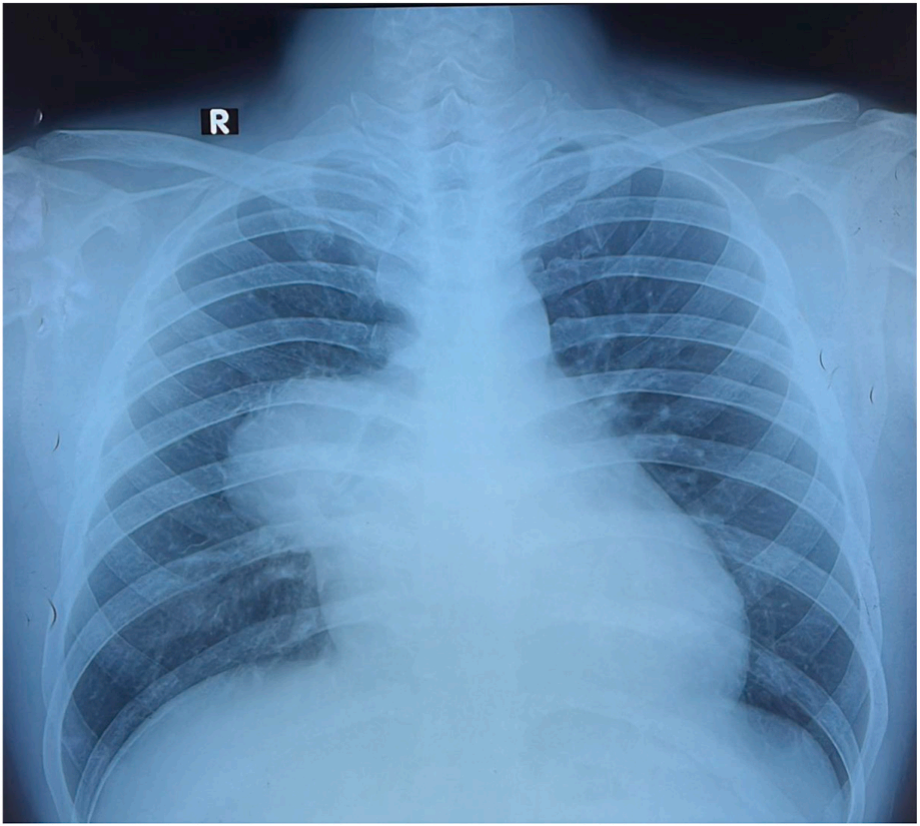
AP chest radiograph.

Chest X-ray showed an anterior mediastinal tumor in the right mediastinum (Fig. 1). Contrast-enhanced CT scan revealed a solid mass with cystic components in the anterior right mediastinum, causing compression of the right pulmonary vein, left atrium, right pulmonary artery, and right middle lobe of the lung. Pericardial effusion and lymphadenopathy were also observed. Possible diagnoses include germ cell tumor (seminoma), thymoma, and lymphoma.

**Fig. 2:**
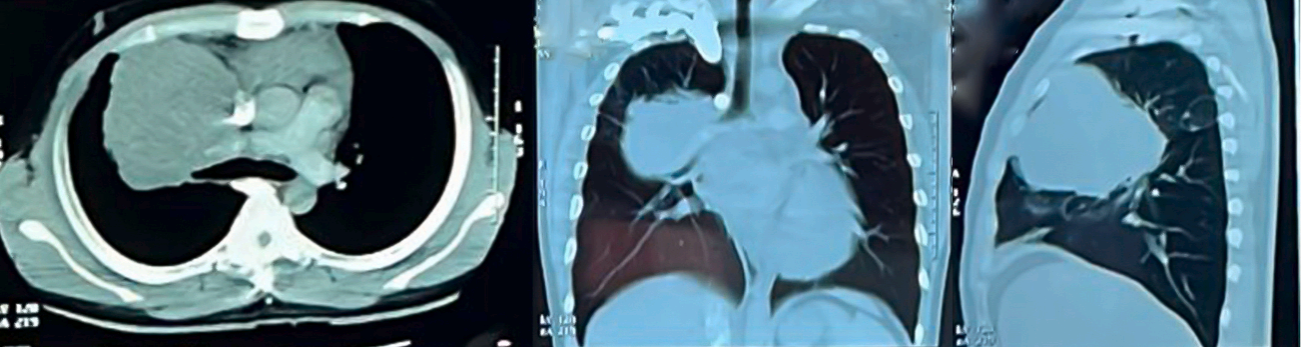
Contrast chest CT image.

Fig. 2 showed a contrast-enhanced CT scan imaging obtained the first time prior to chemotherapy treatment, hence this image was used as the baseline image. This baseline contrast-enhanced CT scan (Fig. 2) revealed an enhancing solid mass in the right anterior-mediastinal and posterior mediastinal region, measuring ±10.5x9.0x15.2 cm. The mass compressed the heart towards the left side, encasing and narrowing the right pulmonary artery, right pulmonary vein, and superior vena cava. Collateral veins were observed in the right supraclavicular region to the right axilla. Lymph nodes were present in the right upper paratracheal (0.7 cm), right lower paratracheal (0.8 cm), and para-aortic (1.2 cm) regions. Fluid density (20 HU) was observed in the pericardial cavity, with a maximum thickness of ±4.2 cm.

**Fig. 3:**
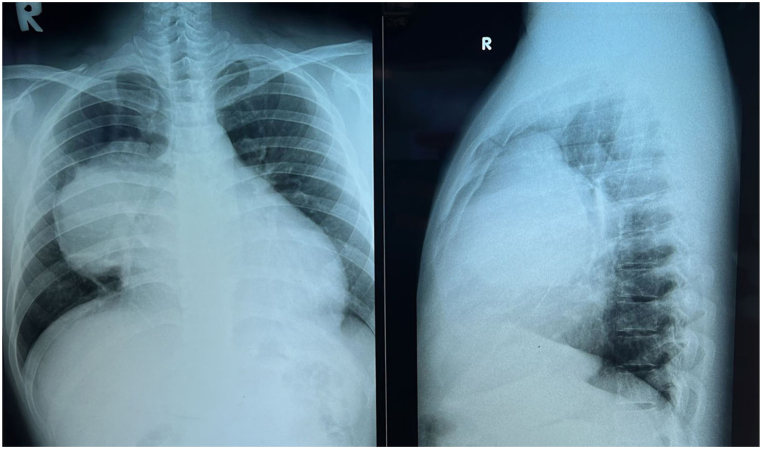
AP and lateral chest x-ray.

AP/Lateral chest x-ray examination results (Fig. 3) showed a mass with regular edge in right hemithorax. Core biopsy with CT guiding and results showed mediastinal mesenchymal spindle tumor. Immunohistochemical examination and CD99, TLE1, and BCL2 results were positive, so the diagnosis of synovial sarcoma was confirmed.

**Fig. 4:**
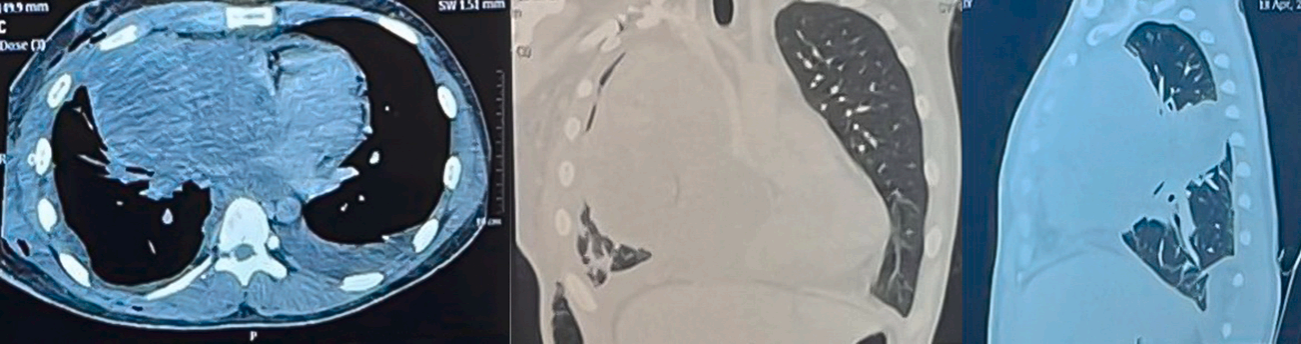
Contrast chest CT.

The debulking procedure through left anterolateral thoracotomy revealed a tumor filling the left thoracic cavity with adhesions to various structures. During the release of tumor boundary, an accidental rupture occurred in the left internal mammary artery, leading to a haemorrhage of ±3000 ml. A contrast-enhanced CT scan (Fig. 4) showed an enhancing solid mass measuring ±14.8x13.9 × 15.8 cm in the anteromedial mediastinum. The mass compressed the heart towards the left side, encasing the right pulmonary artery and right pulmonary vein, and compressed the right main bronchus and its branches. Multiple peritumoral lymph nodes were also observed, with the largest measuring was ±0.6 cm.

The patient's chemotherapy phase consisted of 3 cycles using doxorubicin, ifosfamide, and mesna. Patients receive treatment for chemotherapy side effects of nausea and vomiting, by administering ondancetron tablets. Therapy after chemotherapy is carried out by administering symptomatic therapy with codeine and paracetamol.

**Fig. 5:**
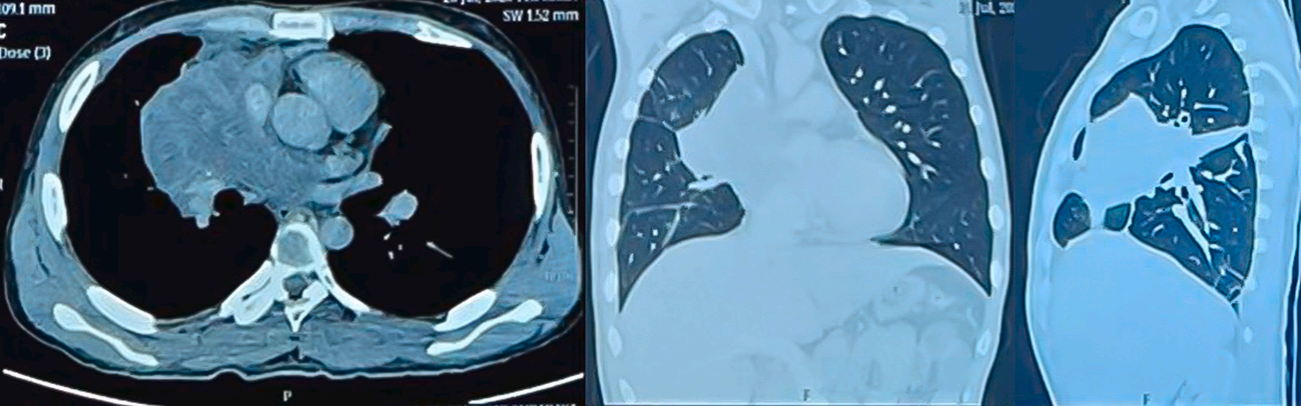
Contrast the CT chest of the patient after 3 cycles of chemotherapy.

The results of chest CT with contrast after chemotherapy (Fig. 5) showed a solid mass in the right anteromedial mediastinum on contrast chest CT with the largest diameter of 11.3 cm, with the conclusion Recist Overall Criteria: Partial Response.

## 3 Discussion

Synovial sarcoma is a rare soft tissue sarcoma, accounting for 8% of all benign tissue tumors. It does not originate from synovial tissue but arises from pluripotent mesenchymal tissue [7]. This condition have multiple potential sites of occurrence, not restricted to the extremities. It mainly occurs in the pleuropulmonary parenchyma in the thorax, while the cor and mediastinum being the less frequently observed sites [4]. Due to the infrequency, diagnostic errors are common. Histologically, synovial sarcoma can exhibit various growth patterns, including monophasic, biphasic, and poorly differentiated types, adding to the diagnostic challenge, especially in uncommon locations [3].

The diagnosis of mediastinal synovial sarcoma is based on clinical symptoms, radiological and pathological examinations, and immunohistochemistry to rule out other primary tumors or sarcoma metastasis. chest pain, cough, and shortness of breath are the most common symptoms this condition, which mainly caused by compression of surrounding organs or tissues [3]. Patients also may have another complaints of hemoptysis, fatigue, weight loss, lung collapse, pleural effusion, pericardial effusion, and superior vena cava obstruction [8].

In addition, the most common initial diagnostic support for this condition is radiological examinations. Opaque lesions of various sizes, sometimes associated with tracheal deviation, mediastinal shift, and pleural effusion may be shown in chest X-rays imaging examination. CT, MRI, and PET/CT imaging are also used to determine the tumor stage, assess resectability, plan surgical resection, and evaluate the response to therapeutic interventions [8]. Mediastinal synovial sarcoma appears as a large, well-defined, heterogeneously enhancing mass with haemorrhage or cystic changes on the CT scan result. Homogeneous or heterogeneous masses with well-defined borders may contain necrotic areas and soft tissue components [9]. In some cases, peripheral calcification has also been described. In advanced stages, invasion and infiltration of surrounding tissues and ipsilateral pleural effusion may occur. In primary pleural synovial sarcoma, adjacent ribs may show sclerotic without chest wall invasion. Conversely, in extrapleural synovial sarcoma, infiltration into adjacent chest wall muscles and erosion of the ribs [10]. Synovial sarcoma is observed as a multilobulated soft tissue mass on MRI test results, exhibiting signal intensity comparable to muscle on T1-weighted images and hyperintensity on T2-weighted imaging, which can be attributed to the presence of cysts and haemorrhage. In, addition, it was observed that the contrast enhancement is heterogenous [[2], [9]] [[2], [9]] It is important for surgeons to be aware that there is little correlation between preoperative radiological findings and operability of mediastinal synovial disease, and the decision for surgical intervention should consider several other factors besides radiological results [8].

In this case report, the patient presented dyspnea, productive cough, chest pain, and weight loss symptoms. The initial chest X-ray image showed a mediastinal mass. A contrast-enhanced CT scan revealed a solid mass in the right anterior-mediastinal and posterior mediastinal region, compressing the heart towards the left side and encasing the right pulmonary artery, right pulmonary vein, and superior vena cava. Collateral veins were observed in the right supraclavicular region to the right axilla, and pericardial effusion was present. A histopathological examination is necessary to confirm the diagnosis of a mediastinal tumor [11]. Synovial sarcoma macroscopically appears as a well-defined mass with variable size, ranging from 5 to 23 cm, with a soft to firm texture and invasion into surrounding tissues [3]. There are four histological variants: monophasic spindle cell, monophasic epithelial, biphasic, and poorly differentiated. The spindle cell monophasic variant is the most common and can be challenging to differentiate from other spindle cell tumors [8].

Definitive diagnosis of synovial sarcoma can be challenging, even after tissue sampling. Immunohistochemistry can be helpful but insufficient as the tumor can histologically resemble other soft tissue tumors. Advanced molecular pathological analysis may be necessary in some cases [12]. Commonly, expressed markers, such as cytokeratin, EMA, bcl-2, S100, synaptophysin, or CD99, are not specific. TLE1 has emerged as a relatively sensitive and specific marker, with a sensitivity range from 82 to 100%. Synovial sarcoma is associated with the translocation of t(X; 18)(p11; q11), resulting in SS18-SSX fusion genes. FISH or RT-PCR is commonly used to detect this rearrangement, providing diagnostic confirmation and representing the most specific and sensitive tool in synovial sarcoma diagnosis [3]. The open biopsy confirmed a spindle mesenchymal tumor with positive immunohistochemistry for CD99 (focal membrane staining), TLE-1 (nuclear staining), and BCL-2 (cytoplasmic staining), consistent with mediastinal synovial sarcoma.

According to the American Joint Committee on Cancer (AJCC) 8th edition, synovial sarcoma is classified based on histological features, tumor size, lymph node involvement, and metastasis [13]. The clinical and radiological features suggest stage IV (G2 T4 N1 M0) synovial sarcoma.

Complete surgical resection is the preferred treatment associated with improved survival. Neoadjuvant chemotherapy and radiotherapy should be considered for inoperable non-metastatic disease, followed by surgical intervention. Adjuvant chemotherapy and radiotherapy should be regarded as part of a multimodal approach for all patients [3]. Studies have shown better overall survival with a combination of surgery and adjuvant therapy than surgery alone [14].

The surgical approach depends on the nature and location of the mediastinal lesion. Minimally invasive approaches are often used for benign, non-invasive masses. More aggressive exposure is required for large and malignant tumors, where complete resection is crucial, but dissection may be challenging [11]. The patient had planned debulking surgery, but during the tumor capsule incision, there was a rupture of the left internal mammary artery with bleeding of ±3000 ml. Therefore, debulking was postponed, and the patient was scheduled for chemotherapy with doxorubicin-ifosfamide.

Synovial sarcoma is often identified as a chemosensitive soft tissue tumor. High-dose ifosfamide has shown a response in all patients with recurrent or metastatic disease in a 1994 publication. Retrospective reviews of advanced synovial sarcoma patients using RECIST criteria found that the combination of doxorubicin and ifosfamide achieved higher response rates than single-agent doxorubicin or ifosfamide [15]. As for mediastinal synovial sarcoma, neoadjuvant chemotherapy with doxorubicin and ifosfamide has been proven to shrink large tumors and facilitate surgical excision [8]. Common side effects of alkylating agent chemotherapy include nausea, vomiting, leukopenia, and thrombocytopenia. Hemorrhagic cystitis can occur with ifosfamide chemotherapy. Adequate fluid hydration and additional mesna therapy based on the patient's weight can help reduce these side effects [16].

Adjuvant chemotherapy and radiation therapy should be considered as part of a multimodal approach for all patients. External beam radiation therapy (EBRT) is commonly used for inoperable, metastatic, and incompletely debulked primary mediastinal synovial sarcoma. EBRT has been shown to offer local disease control and overall survival benefits in these patient groups [3,8].

Based on body surface area, the patient received 3 cycles of chemotherapy with doxorubicin, ifosfamide, and mesna. Side effects included nausea, managed with ondansetron, and occasional non-vomiting nausea, improved with ondansetron tablets. Blood tests and electrolyte levels were within normal limits.

The prognosis for mediastinal synovial sarcoma is poor, with an estimated 5-year overall survival rate of 35.7%. Poor clinical and microscopic prognostic factors include age >20 years, male gender, positive surgical margins (incomplete resection), tumor size >5 cm, neurovascular infiltration, extensive tumor necrosis, high mitotic count (>10/10 HPF), high-grade undifferentiated histology, biphasic variant, SYT-SSX1 chromosomal translocation, and distant metastasis [8]. The patient, a male, had a tumor size of ±14.8x13.9 × 15.8 cm on a CT scan. Histopathological examination showed 4/10 HPF mitosis, and immunohistochemistry confirmed synovial sarcoma. Resection attempts failed, and the patient underwent 3 cycles of adjuvant chemotherapy with doxorubicin, ifosfamide, and mesna. Evaluation after 3 cycles showed an enhancing solid mass in the right anteromedial mediastinum with a diameter of approximately 11.3 cm. Bilateral pleural effusion was present on the initial contrast-enhanced CT scan but not on the second scan. Based on RECIST's overall criteria, the conclusion was a partial response, with dubia and malam as a prognosis.

## 4 Conclusion

A 23-year-old male with mediastinal synovial sarcoma presented with symptoms of dyspnea, cough, chest pain, and weight loss. Chest X-ray showed a mass in the anterior mediastinum, and a contrast-enhanced CT scan revealed a large enhancing solid mass compressing the heart, encasing and narrowing the right pulmonary artery, right pulmonary veins, and superior vena cava. Pericardial effusion was also present. Histopathological and immunohistochemical examination confirmed the diagnosis of synovial sarcoma. Debulking surgery was unsuccessful, and the patient received 3 cycles of adjuvant chemotherapy with doxorubicin, ifosfamide, and mesna. Evaluation after 3 cycles showed a decrease in tumor size and resolution of pleural effusion, indicating a partial response according to RECIST criteria.

## Consent

Written informed consent was obtained from the patient to publish this case report and accompanying images. A copy of the written consent is available for review by the Editor-in Chief of this journal on request.

## Sources of funding

This research received no external funding.

## Ethical approval

Ethical approval was not required for this case report, however written informed consent was obtained from the patient and is available for review under request.

## Research registration

Not applicable.

## Authorship contribution statement

INI, IAM, DW conceptualized and investigated the case; INI drafted the manuscript and curated the data; IAM, DW supervising the manuscript; INI searched the literature data; IAM, DW reviewed and revised the manuscript.

## CRediT authorship contribution statement

**Intan Nurani Indrajanu:** Conceptualization, Data curation, Investigation, Writing – original draft. **Isnin Anang Marhana:** Conceptualization, Investigation, Supervision, Validation, Writing – review & editing. **Dwi Wahyu Indrawanto:** Conceptualization, Investigation, Supervision, Validation, Writing – review & editing.

## Declaration of competing interest

All authors declare no conflicts of interest.

## References

[1] Blay J.Y., von Mehren M., Jones R.L. (2023). Synovial sarcoma: characteristics, challenges, and evolving therapeutic strategies. ESMO Open.

[2] Ouji M., Souissi S., Koubaa M., Boussetta Mezghani (2015). Primary synovial sarcoma of the Mediastinum: a case report and literature review. J. Clin. Case Rep..

[3] Syred K., Weissferdt A. (2020). Primary mediastinal sinovial sarkomas. Mediastinum.

[4] Terra S.B.S.P., Aesif S.W., Maleszewski J.J., Folpe A.L., Boland J.M. (2018). Mediastinal synovial sarcoma: clinicopathologic analysis of 21 cases with molecular confirmation. Am. J. Surg. Pathol..

[5] Fitra A.F., Kloping Y.P., Djatisoesanto W., Hakim L. (2022). Doxorubicin and ifosfamide for recurrent renal synovial sarcoma: the first case report in Indonesia. Int J Surg Case Rep.

[6] He H., Yang L., Peng Y. (2021). The value of multidisciplinary team (MDT) management in the diagnosis and treatment of primary intrathoracic synovial sarcomas: a single-center experience. J. Thorac. Dis..

[7] Mrabet F.Z., El Ouazzani H., El Akkari L., Hammi S., Bourkadi J.E., Zouaidia F. (2018). Primary pleuropulmonary synovial sarcoma: a case. Case Rep Pulmonol.

[8] Abu-Zaid A., AlNajjar A., Alotaibi S. (2018). Huge primary mediastinal synovial sarcoma fully occupying the right hemithorax. J. Cancer Res. Therapeut..

[9] Wang D.J., Alwafi L., Pritchett S.L., Wehrli B.M., Spouge A.R.I. (2021). The imaging spectrum of synovial sarcomas: a pictorial review from a single-centre tertiary referral institution. Can. Assoc. Radiol. J..

[10] De Paoli L., Quaia E., Poillucci G., Gennari A., Cova M.A. (2015). Imaging characteristics of pleural tumors. Insights into Imaging.

[11] Hazzard C., Kaufman A., Flores R., Pass H.I., Ball D., Scagliotti G.V. (2018). IASLC Thoracic Oncology.

[12] Khamaysi I., Naroditsky I., Malkin L. (2021). Primary synovial sarcoma of the mediastinum: a rare tumor diagnosed by endoscopic ultrasound-fine needle biopsy (EUS-FNB)-Cytomorphologic, immunohistochemical, and molecular analysis. Clin J Gastroenterol.

[13] Tanaka K., Ozaki T. (2019). New TNM classification (AJCC eighth edition) of bone and soft tissue sarcomas: JCOG Bone and Soft Tissue Tumor Study Group. Jpn. J. Clin. Oncol..

[14] Engelhardt K.E., DeCamp M.M., Yang A.D., Bilimoria K.Y., Odell D.D. (2018). Treatment approaches and outcomes for primary mediastinal sarcoma: analysis of 976 patients. Ann. Thorac. Surg..

[15] Riedel R.F., Jones R.L., Italiano A. (2018). Systemic anti-cancer therapy in synovial sarcoma: a systematic review. Cancers.

[16] Yuliza Wulandari L. (2021). Rare case of mediastinal sinovial sarkoma in an Indonesian woman. Med. Leg. Update.

